# Dynamic microenvironments for bone organoids: spatiotemporal regulation of angiogenic-osteogenic coupling through advanced hydrogel design

**DOI:** 10.3389/fbioe.2026.1958191

**Published:** 2026-09-23

**Authors:** Sen Liu, Lang Tian, Yayue Zheng, Yingxin Chen, Yangjun Liu, Xiujie Wen

**Affiliations:** 1 Department of Orthodontics, The Affiliated Stomatological Hospital, Southwest Medical University, Luzhou, China; 2 Department of Orthodontics, Chongqing Ruitai Stomatological Hospital, Chongqing, China

**Keywords:** angiogenic-osteogenic coupling, bone organoids, dynamic hydrogels, spatiotemporal regulation, vascularized bone regeneration

## Abstract

Bone organoids are promising platforms for studying skeletal development, disease and regeneration, but their broader application is limited by inadequate vascularization and the difficulty of recapitulating the evolving native bone microenvironment. *In vivo*, angiogenesis and osteogenesis are tightly coupled processes that overlap spatially and temporally, with their relative dominance shifting during tissue development and repair. Conventional hydrogels are generally too static to reproduce this dynamic interplay. This Review examines how dynamic hydrogels can provide adaptive biochemical, mechanical and structural cues to direct angiogenic–osteogenic coupling in bone organoids. Tunable degradation, viscoelasticity, stimulus responsiveness, and release kinetics may promote an early angiogenic bias while supporting overlapping and progressively enhanced osteogenic differentiation, extracellular matrix deposition, and mineral maturation. The Review distinguishes direct evidence from bone organoid or organoid-like models from indirect evidence derived from related bone tissue-engineering systems. We discuss experimentally demonstrated and proposed strategies, including temporally biased delivery systems, stress-relaxing networks, piezoelectric hydrogels, and spatial patterning, and assess their potential effects on vascular stability, lineage specification, and host integration. We also outline key challenges facing the translation of dynamic hydrogel-based bone organoids into regenerative grafts. By recapitulating the developmental logic of bone formation, dynamic hydrogels may advance the engineering of vascularized bone tissues.

## 1 Introduction

Critical-sized bone defects caused by trauma, tumor resection, or congenital deformities remain a major challenge in orthopedic and craniofacial surgery (Koolen et al., 2020; Yu et al., 2020; Wang et al., 2025). Although autologous bone grafting is the gold standard, it is limited by donor tissue availability and harvest-site morbidity (Li et al., 2024; Qiao et al., 2022; Minetti et al., 2023). Allografts and synthetic substitutes often fail to achieve consistent osseointegration and carry risks of immune rejection or disease transmission (Roberts and Rosenbaum, 2012; Giannoudis et al., 2005). Therefore, advanced regenerative strategies are needed.

Bone tissue engineering has traditionally relied on scaffolds, cells, and growth factors, but conventional scaffolds often provide static architectures that do not adequately reproduce the dynamic microenvironment of native bone (Place et al., 2009; Lutolf and Hubbell, 2005; Hollister, 2005). Bone organoids have emerged as an alternative. In this Review, bone organoids are defined as three-dimensional, multicellular, self-organizing constructs derived from stem, progenitor, or tissue-derived cells that reproduce selected features of bone tissue, such as osteogenic differentiation, extracellular matrix deposition, and mineralization (Zhu et al., 2025; Bai et al., 2024; Unagolla and Jayasuriya, 2022).

Because the terms “bone organoid,” “bone-like organoid,” and “organoid-like bone construct” are used inconsistently, we distinguish between direct and indirect evidence. Studies using bone organoids or organoid-like bone constructs are considered direct evidence, whereas studies based on MSC cultures, vascularized bone-regeneration constructs, cartilage or endochondral models, callus-like constructs, wound-healing models, and general hydrogel systems are considered indirect evidence. Findings from these related systems inform hydrogel design but are not interpreted as direct evidence of bone organoid formation, maturation, vascularization, or integration.

A major bottleneck persists: insufficient vascularization. Bone organoids, especially those of clinically relevant sizes, lack a functional vascular network (Faeed et al., 2024; Zhang X. et al., 2025). The diffusion limit of oxygen and nutrients in dense three-dimensional cultures is typically around 100–200 μm (Lee et al., 2020; Griffith and Swartz, 2006). Without an intrinsic blood supply, cells in the core of large organoids experience hypoxia, nutrient deprivation, and metabolic waste accumulation, leading to necrosis, impaired maturation, and poor host integration (Wang et al., 2026). Achieving robust and functional vascularization is therefore a critical prerequisite for translating bone organoids into clinical practice.

In native bone development and repair, angiogenesis and osteogenesis are tightly coupled in a precise spatiotemporal sequence (Kusumbe et al., 2014; Kanczler and Oreffo, 2008; Ramasamy et al., 2016). During endochondral or intramembranous ossification, blood vessels invade nascent tissue first, establishing perfusion routes that deliver oxygen, nutrients, osteoprogenitor cells, and signaling molecules (Gerber et al., 1999a; Maes et al., 2010). Following vascular invasion, osteoblasts deposit and mineralize bone matrix along the pre-existing vascular network (Kusumbe et al., 2014). This orderly angiogenic–osteogenic cascade is essential for forming mature bone. Recapitulating this coupling *in vitro* is therefore a key design principle for engineering functional bone organoids.

Conventional hydrogel systems generally offer static physicochemical cues (Lutolf and Hubbell, 2005; Burdick and Murphy, 2012). Their permanently crosslinked networks often restrict cellular remodeling and limit spatiotemporal presentation of bioactive factors. When angiogenic and osteogenic factors are simply co-encapsulated, they tend to be released simultaneously, failing to mimic the sequential signaling required for proper bone morphogenesis (Kempen et al., 2009; Patel et al., 2008). This limitation underscores the need for biomaterial platforms that can dynamically orchestrate cell behavior.

Dynamic hydrogels have emerged as a powerful solution. They are characterized by their ability to undergo changes in mechanical properties, degradation, or ligand presentation over time, either through passive mechanisms (e.g., hydrolytic or enzymatic degradation) or active responsiveness to external stimuli (e.g., ultrasound, light, or pH) (Rosales and Anseth, 2016; Kloxin et al., 2009; Lutolf et al., 2003; DeForest and Anseth, 2011). Key design features include tunable stress relaxation, programmed degradation rates, and spatiotemporally controlled release of multiple bioactive molecules. These features enable the hydrogel to evolve with the developing organoid, initially providing a pro-angiogenic environment and later transitioning to an osteoinductive niche (Kempen et al., 2009). Dynamic hydrogels are therefore uniquely positioned to recapitulate angiogenic–osteogenic coupling in a temporally regulated manner.

Several studies have investigated temporal coordination of angiogenic and osteogenic signals. Sequential delivery of vascular endothelial growth factor (VEGF) followed by bone morphogenetic protein 2 (BMP-2) has been reported to improve vascularized bone regeneration compared with simultaneous delivery (Kempen et al., 2009; Patel et al., 2008). These findings provide indirect design rationale for bone organoid engineering but do not demonstrate that the same sequence has been validated in a bone organoid model. Hydrogel stress relaxation and matrix elasticity should be discussed separately; these properties have been associated with changes in cell spreading, matrix remodeling, stem-cell behavior, and osteogenic differentiation in related models (Chaudhuri et al., 2016; Huebsch et al., 2015). Hypoxia-related strategies targeting hypoxia-inducible factor (HIF) signaling, including pharmacological inhibition of prolyl hydroxylases, have been investigated in skeletal-development and tissue-engineering models (Wang et al., 2007; Schipani et al., 2009; Epstein et al., 2001; Elvidge et al., 2006). These studies support the biological plausibility of coupling hypoxia-related angiogenic signaling with osteogenesis, but their direct application in bone organoid systems remains to be established.

The central challenge in engineering bone organoids is not simply to add vascular components, but to reproduce the developmental sequence in which vascularization precedes and enables osteogenesis (Kusumbe et al., 2014). This requires biomaterials that are not merely permissive, but actively instructive over time. Dynamic hydrogels offer such a platform by integrating programmed degradation, viscoelastic remodeling, and spatiotemporally controlled presentation of biochemical and mechanical cues (Figure 1). The following sections discuss design principles, representative material systems, and material–biology relationships that govern angiogenic–osteogenic coupling, followed by current limitations and future directions.

**FIGURE 1:**
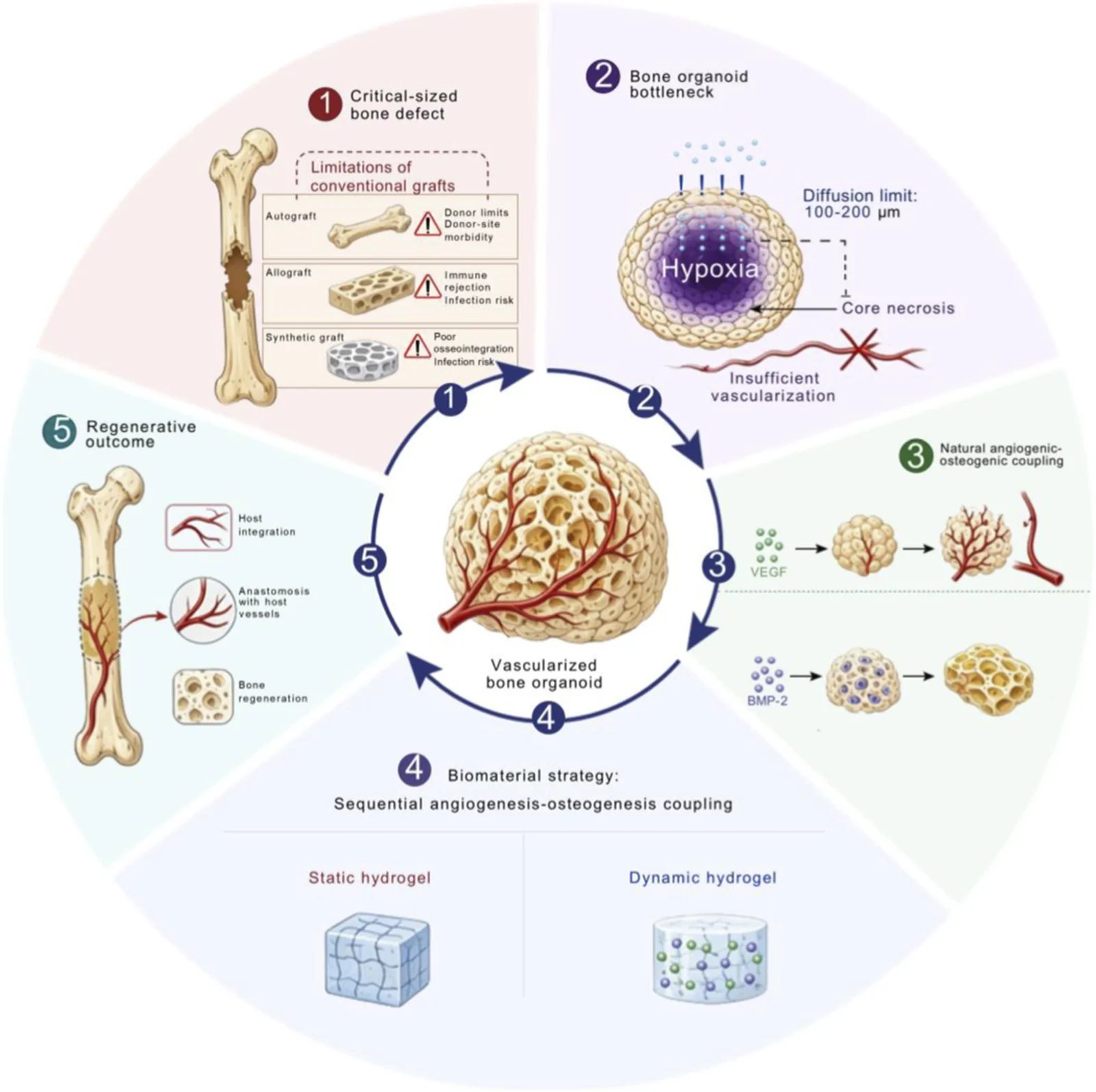
Conceptual framework for dynamic hydrogel design in vascularized bone-organoid engineering.

This Review focuses on dynamic hydrogel features relevant to vascularized bone-organoid engineering, including time-dependent mechanics, cell-mediated matrix remodeling, programmed degradation, stimulus responsiveness, spatial patterning, and temporally biased presentation of biochemical cues. It does not treat every mineralizing hydrogel construct as a bone organoid. Instead, the literature is organized according to the evidence hierarchy described above. Direct evidence is discussed when a dynamic hydrogel has been evaluated in a bone organoid or organoid-like bone model; adjacent-system evidence is used to explain mechanisms from MSC cultures, vascularized bone-regeneration models, cartilage or endochondral systems, wound-healing models, or general biomaterial platforms; and proposed design concepts are presented as testable hypotheses.

## 2 Challenges in vascularizing bone organoids and the rationale for spatiotemporal control

### 2.1 Diffusion barriers and central necrosis

Vascularization remains a persistent barrier to translating bone organoids into implantable regenerative constructs (Novosel et al., 2011). Although bone organoids can reproduce aspects of osteogenic differentiation, extracellular matrix deposition, and mineralization, these processes are strongly constrained by mass transport (Griffith and Swartz, 2006). In dense three-dimensional tissues, oxygen and nutrients diffuse only over limited distances before being consumed by metabolically active cells (Griffith and Swartz, 2006; Folkman and Hochberg, 1973). As organoids increase in size, the central region becomes progressively isolated from the culture medium, leading to steep gradients of oxygen, glucose, growth factors, pH, and metabolic waste (Griffith and Swartz, 2006; Lancaster and Knoblich, 2014; McMurtrey, 2016). The practical diffusion distance for oxygen in most mammalian 3D cultures is commonly estimated to be 100–200 μm, depending on cell density, oxygen consumption rate, matrix permeability, and culture geometry (Griffith and Swartz, 2006; Folkman and Hochberg, 1973; McMurtrey, 2016). Beyond this range, cells in the organoid core are prone to hypoxia, growth arrest, apoptosis, or necrosis (Volkmer et al., 2008; Takebe et al., 2013).

Dynamic hydrogels may improve matrix remodeling, cell organization, and the formation of vascular-like structures; however, a dynamic hydrogel alone does not eliminate the diffusion limit in a construct of a given size under static culture conditions. Self-assembled vascular networks formed *in vitro* are frequently nonperfused and cannot be assumed to relieve core hypoxia before active perfusion or host-vessel anastomosis. The potential value of a dynamic hydrogel is indirect: it may create a more permissive microenvironment that supports vascular organization, improves cell survival during early culture, and accelerates host-driven perfusion after implantation. Direct relief of oxygen and nutrient limitation requires perfusion, reduced diffusion distance, internal channels, active fluid flow, or sufficiently rapid inosculation with host vasculature (Lee et al., 2020; Griffith and Swartz, 2006; Novosel et al., 2011; Folkman and Hochberg, 1973; Volkmer et al., 2008; Chen et al., 2009; Laschke and Menger, 2016).

Osteogenesis itself is energy intensive; bone progenitor cells need to proliferate, differentiate, synthesize collagen-rich matrix, and support mineral deposition, all of which require sufficient oxygen and nutrients (Dirckx et al., 2018; Esen et al., 2013; Long, 2011). Transient or moderate hypoxia may contribute to developmental signaling and HIF-dependent angiogenic responses, whereas prolonged or severe hypoxia can impair cell survival, matrix organization, and organoid maturation (Wang et al., 2007; Schipani et al., 2009). Uncontrolled core hypoxia decreases cell activity, disrupts matrix tissue structure, and may form necrotic regions while weakening mechanical strength (Griffith and Swartz, 2006; McMurtrey, 2016). After implantation, survival of a prevascularized construct depends on the timing and quality of host-vessel invasion, inosculation, and anastomosis. Preformed vascular-like networks may accelerate host-driven perfusion, but they generally do not make the graft independent of host circulation. The rate of rescue of the construct core is determined by construct size, vascular-network density and maturity, endothelial–perivascular interactions, matrix permeability, implantation site, inflammatory response, and time required for functional connection to host vessels (Chen et al., 2009; Laschke and Menger, 2016; Rouwkema et al., 2008). If anastomosis is delayed, hypoxic regions may undergo irreversible injury before perfusion is established. Pre-existing vascular organization, adequate matrix permeability, and timely host anastomosis are therefore all important for improving organoid survival and integration.

### 2.2 Limits of conventional prevascularization strategies

Endothelial–mesenchymal co-culture is one of the most widely used approaches (Levenberg et al., 2005; Au et al., 2008). Endothelial cells (e.g., HUVECs or endothelial colony-forming cells) are combined with mesenchymal stem cells or osteoprogenitors in a 3D matrix (Esen et al., 2013; Fedorovich et al., 2009). Through paracrine signaling and cell–cell interactions, endothelial cells form capillary-like networks, while mesenchymal cells provide perivascular support and contribute to osteogenic differentiation (Kanczler and Oreffo, 2008; Au et al., 2008; Ghajar et al., 2010). However, these networks are often immature, poorly perfusable, and spatially disorganized; their stability after implantation depends strongly on rapid anastomosis with host vasculature, which is not guaranteed (Long, 2011).

Growth factor delivery represents another common strategy. VEGF, fibroblast growth factor 2 (FGF-2), platelet-derived growth factor BB (PDGF-BB), and angiopoietins have been used to stimulate endothelial migration, sprouting, and lumen formation (Carmeliet, 2003; Gerhardt et al., 2003; Lindahl et al., 1997; Ferrara et al., 2003). However, simple bolus delivery or uncontrolled release frequently produces short-lived gradients and nonphysiological concentrations (Burdick and Murphy, 2012; Richardson et al., 2001; Silva and Mooney, 2007). Excessive or poorly timed VEGF exposure can generate leaky, unstable, or aberrant vessels rather than mature vascular networks. High or poorly localized VEGF exposure can produce unstable or leaky vessels, and the translational performance of VEGF-based therapies has been limited by dose, distribution, and vessel-maturation issues (Ozawa et al., 2004; Dor et al., 2002; Jain, 2003). Conversely, insufficient exposure fails to support endothelial invasion (Gerhardt et al., 2003; Gerber et al., 1999b). Similar problems arise when angiogenic and osteogenic cues are co-delivered without temporal control (Kempen et al., 2009; Patel et al., 2008).

Bioprinting, sacrificial templating, and microfluidic platforms provide advanced options for building vascular channels (Kolesky et al., 2014; Kolesky et al., 2016; Miller et al., 2012; Bhatia and Ingber, 2014). These technologies improve architectural control and perfusion, but printed channels may not recapitulate capillary-scale remodeling, and microfluidic systems can be difficult to translate into clinically implantable grafts (Long, 2011; Kolesky et al., 2016; Bhatia and Ingber, 2014; Grigo et al., 2019). Vascularization is not only a structural engineering problem; it is a dynamic biological process requiring temporal coordination among endothelial cells, osteoprogenitors, immune cells, matrix remodeling, and soluble signaling.

#### 2.2.1 Perfusion-assisted vascularization

Perfusion should be considered a complementary strategy. Perfusion bioreactors and microfluidic culture systems can directly reduce transport limitations during *in vitro* culture by supplying oxygen and nutrients through controlled convective flow. In contrast, static culture of a hydrogel containing self-assembled vascular-like networks does not provide equivalent transport. Microfluidic devices can establish defined pressure gradients, shear stress, and soluble-factor gradients, although their scale-up and integration with implantable grafts remain challenging (Kolesky et al., 2014; Kolesky et al., 2016; Miller et al., 2012; Bhatia and Ingber, 2014; Grigo et al., 2019; Rouwkema et al., 2008). Perfusion-assisted culture is particularly important when organoids exceed the effective diffusion distance or when mineralization increases matrix density. Relevant outcome measures include oxygen concentration, flow rate, pressure drop, endothelial barrier integrity, lumen patency, shear-responsive gene expression, and viability across the construct. These parameters should be reported separately from vascular markers, because endothelial organization alone does not demonstrate functional perfusion.

#### 2.2.2 Host inosculation and anastomosis

Prevascularization accelerates, rather than replaces, host-driven perfusion. The therapeutic value of preformed networks depends on their ability to connect to host vessels within a time window that preserves organoid viability. This process is influenced by endothelial maturity, perivascular support, vessel diameter, matrix degradability, implantation site, inflammatory signaling, and host capillary density (Gilbert-Honick and Grayson, 2020; Esen et al., 2013; Long, 2011; Rouwkema et al., 2008). Future studies should distinguish among endothelial network formation *in vitro*, perfusion under bioreactor conditions, host-vessel invasion, inosculation, and durable blood-flow establishment after implantation.

### 2.3 Angiogenesis–osteogenesis coupling as a developmental principle

In both endochondral and intramembranous ossification, vascular invasion and bone formation are closely related, but blood vessels do more than transport oxygen and nutrients (Gerber et al., 1999a; Percival and Richtsmeier, 2013; Saran et al., 2014). They release signaling molecules, guide directional transport of progenitor cells, and establish local microenvironments that affect the spatial distribution of matrix deposition (Kusumbe et al., 2014; Maes et al., 2010). Among skeletal vascular subtypes, type H vessels, characterized by high expression of CD31 and endomucin, provide a strong biological rationale for coupling angiogenesis to osteogenesis. Type H endothelial cells are enriched near osteoprogenitor populations and are regulated by HIF, VEGF, Notch, and Dll4 signaling (Kusumbe et al., 2014; Ramasamy et al., 2016; Langen et al., 2017; Ramasamy et al., 2014). Their association with osteoprogenitors is not merely anatomical; endothelial-derived and perivascular signals can influence progenitor recruitment, proliferation, and osteogenic differentiation. A vascularized bone organoid should therefore be evaluated not only by total vessel density but also by endothelial phenotype, vessel subtype, perivascular organization, and spatial proximity to osteogenic progenitors.

Physiological bone undergoes continuous remodeling through coupled osteoblast-mediated deposition and osteoclast-mediated resorption, and this remodeling axis is increasingly recognized as an integral component of angiogenic–osteogenic coupling (Robling et al., 2006). Preosteoclast-derived platelet-derived growth factor BB (PDGF-BB) promotes type H vessel formation and recruits osteoprogenitors (Kusumbe et al., 2014; Ramasamy et al., 2016), while osteoclast-lineage cells also influence matrix turnover, mineral release, and local signaling. A bone organoid that deposits mineralized matrix but cannot undergo controlled resorption remains an incomplete model of skeletal tissue. Future systems should therefore incorporate osteoclast precursors or osteoclast-like cells, together with receptor activator of nuclear factor-κB ligand (RANKL) and macrophage colony-stimulating factor (M-CSF), using resorbable substrates such as calcium phosphate or dentin matrix. Relevant endpoints include tartrate-resistant acid phosphatase (TRAP) activity, cathepsin K expression, resorption pit formation, and the balance between mineral deposition and resorption. This extends the design framework from angiogenic–osteogenic coupling to angiogenic–osteogenic–remodeling coupling.

Thus, the central design question is not only “how can vessels be added to bone organoids?” but also “how can vascular and osteogenic programs be coordinated with appropriate spatial and temporal bias?” A preformed vascular-like network without osteogenic integration may not support bone regeneration; a mineralized organoid without internal vascular access will remain size-limited. The goal is not to impose a strictly separated sequence, but to establish an early angiogenic bias that supports vascular organization while allowing osteogenic differentiation, matrix deposition, and mineralization to proceed in overlapping and progressively coordinated phases (Kusumbe et al., 2014; Gerber et al., 1999a; Maes et al., 2010).

### 2.4 The marrow and immune microenvironment

Bone organoids intended to model mature skeletal tissue should be considered in relation to the marrow niche, which includes osteogenic stromal cells, endothelial cells, perivascular cells, hematopoietic progenitors, immune cells, adipogenic populations, extracellular matrix, and gradients of oxygen and metabolites. Endothelial and stromal populations do not function independently of hematopoietic or immune signals.

Macrophages are central mediators of both angiogenesis and the host response to implanted hydrogels. Their polarization state—often simplified as M1 versus M2—can either promote or inhibit vascular network stabilization, matrix remodeling, and osteogenic differentiation. Hydrogel stiffness, ligand presentation, degradation chemistry, and released mineral or polymeric fragments can all influence macrophage recruitment and polarization (Liu et al., 2023; Kuo et al., 2025). For example, rapidly degrading hydrogels that generate acidic by-products may favor a pro-inflammatory phenotype, whereas controlled degradation and calcium phosphate release may support a more reparative phenotype. Therefore, bone organoid studies should report macrophage markers (e.g., CD86, CD206), inflammatory cytokines (TNF-α, IL-1β, IL-10), and foreign-body responses alongside vascular and osteogenic outcomes. Immunomodulatory cues, such as IL-4, IL-10, or specific material chemistries, could be incorporated in a temporally biased manner to promote a supportive immune microenvironment during early vascularization and later matrix maturation.

The marrow niche comprises hematopoietic stem and progenitor cells, endothelial cells, perivascular mesenchymal stromal cells, adipocytes, and extracellular matrix components that jointly regulate hematopoiesis and skeletal homeostasis. Endothelial cells in the marrow, particularly type H vessels, are in close contact with perivascular osteoprogenitors and hematopoietic cells, creating a functional unit that couples bone formation, vascular maintenance, and blood cell production. Bone organoid models that include a marrow-like compartment could therefore provide a more complete platform for studying skeletal diseases such as leukemia, bone metastasis, or marrow failure syndromes. A minimal osteogenic organoid need not contain a full hematopoietic compartment, but studies claiming marrow-like organization should demonstrate hematopoietic cell maintenance, perivascular localization, and the presence of key niche factors such as CXCL12 and SCF.

The inclusion of hematopoietic or immune cells should be guided by the biological question; a minimal osteogenic organoid may not require a complete marrow compartment, whereas a marrow-containing, fracture-callus, or disease-model organoid may require hematopoietic, immune, and osteoclast-lineage components. This distinction should be stated explicitly when comparing different organoid systems.

### 2.5 Shortcomings of conventional static hydrogel matrices

Conventional hydrogels provide hydrated environments, tunable stiffness, and biochemical modification (Drury and Mooney, 2003; Tibbitt and Anseth, 2009; Caliari and Burdick, 2016; Buwalda et al., 2014), but many are essentially static after gelation. Crosslinking density, ligand presentation, degradation profile, and growth factor release kinetics are largely fixed at fabrication (Lutolf and Hubbell, 2005; Lutolf et al., 2003; Mammoto et al., 2011). This static character conflicts with the dynamic nature of bone development (Kanczler and Oreffo, 2008). Cells need to spread, migrate, remodel matrix, establish multicellular contacts, and respond to changing signals. If the matrix is too restrictive, endothelial sprouting and osteoprogenitor remodeling are impaired (Mammoto et al., 2011; Rouwkema et al., 2008); if bioactive cues are released too rapidly or without sequence, the system loses developmental fidelity (Kempen et al., 2009; Patel et al., 2008).

Simultaneous encapsulation of VEGF and BMP-2 may appear attractive, but it often fails to capture the natural order of bone repair (Kanczler and Oreffo, 2008; Kempen et al., 2009). An early relative predominance of VEGF may help recruit and organize endothelial networks, whereas osteogenic cues such as BMP-2, mineral components, or osteoinductive peptides may become increasingly important as the vascular and stromal microenvironment develops (Kanczler and Oreffo, 2008; Kempen et al., 2009; Patel et al., 2008). This does not require complete temporal separation; rather, excessive or premature osteogenic stimulation may compete with endothelial morphogenesis, and uncontrolled mineralization may reduce matrix permeability before adequate vascular organization (Kanczler and Oreffo, 2008; Rouwkema et al., 2008).

Matrix mechanics cannot be reduced to stiffness alone. Cells respond to combined elastic modulus, stress relaxation, creep, plasticity, ligand mobility, degradability, and cell-mediated remodeling (Burdick and Murphy, 2012; Chaudhuri et al., 2016; Chaudhuri et al., 2015; Auger et al., 2013; Chaudhuri et al., 2020). Rapid stress relaxation can permit cell spreading and matrix deformation even when initial elastic modulus is unchanged (Chaudhuri et al., 2015), but the biological effect depends on initial modulus, cell type, ligand presentation, and culture stage. Therefore, dynamic mechanical design should specify both the initial mechanical state and its time-dependent evolution. The dynamic hydrogel network can also regulate blood vessel morphogenesis by changing endothelial cell contractility, integrin distribution, signaling pathways, and extracellular matrix remodeling (Rouwkema et al., 2008; Wei et al., 2023). These findings support the need for materials that provide biochemical signals and mechanical capacity to change over time (Burdick and Murphy, 2012; Kloxin et al., 2009; DeForest and Anseth, 2011).

### 2.6 Spatiotemporal control as a biomaterials design rule

Spatiotemporal control converts hydrogels from passive cell carriers into instructive developmental microenvironments (Lutolf and Hubbell, 2005; Burdick and Murphy, 2012; DeForest and Anseth, 2011). Temporally, the material should establish an early bias toward endothelial survival, migration, lumen formation, and network stabilization while preserving conditions that allow osteogenic differentiation to begin and continue. As the organoid matures, osteogenic differentiation, matrix deposition, and mineral maturation may become increasingly dominant, although vascular remodeling and endothelial–stromal signaling persist. Spatially, it should organize gradients of oxygen, angiogenic factors, osteogenic cues, adhesive ligands, and mechanical properties so that different regions mature in a coordinated manner (Griffith and Swartz, 2006; Rouwkema et al., 2008).

Dynamic hydrogels are well suited to this task. Degradation-mediated release can program sequential delivery of bioactive factors: a faster-degrading compartment may release VEGF or DMOG during the early phase, while a slower-degrading compartment delivers BMP-2, nano-hydroxyapatite, or osteogenic peptides over a longer period (Kempen et al., 2009; Patel et al., 2008). Viscoelastic hydrogels allow cells to remodel their local environment, essential for endothelial sprouting and mesenchymal condensation. Stimuli-responsive hydrogels enable on-demand changes in stiffness, degradation, ligand exposure, or factor release in response to light, ultrasound, temperature, enzymatic activity, or pH (Kloxin et al., 2009; DeForest and Anseth, 2011). The value of this approach lies in encoding biological sequence into material structure: an early phase matrix may be more permissive, remodelable, and enriched with angiogenic signals; later maturation may acquire greater osteoinductive, mineralization-promoting, and mechanically supportive characteristics (Kempen et al., 2009; Chaudhuri et al., 2016; Huebsch et al., 2015). This evolution reflects a changing balance between vascular organization and bone matrix formation rather than a complete transition from one process to another.

### 2.7 Design criteria for vascularized bone organoids

A rational hydrogel system for vascularized bone organoids should: (1) support endothelial network formation without compromising osteoprogenitor viability, while allowing angiogenic and osteogenic activities to overlap (Miller et al., 2012; Rouwkema et al., 2008); (2) maintain sufficient permeability for oxygen and nutrient transport before functional vascularization (Griffith and Swartz, 2006; Rouwkema et al., 2008); (3) provide degradation or remodeling pathways that enable cell invasion, spreading, and matrix reorganization (Lutolf et al., 2003; Chaudhuri et al., 2016); (4) establish temporally biased presentation of angiogenic and osteogenic signals rather than a single simultaneous burst or complete temporal separation (Kempen et al., 2009; Patel et al., 2008; Richardson et al., 2001); and (5) exhibit mechanical properties that evolve with tissue maturation, providing early cellular permissiveness and later structural support (Burdick and Murphy, 2012; Kloxin et al., 2009; DeForest and Anseth, 2011). Quantitative design is also needed: parameters such as degradation half-life, mesh size, storage modulus, stress-relaxation time, ligand density, factor dose, and release kinetics should be linked to measurable biological outcomes. However, direct comparison is currently limited by differences in hydrogel composition, testing conditions, cell sources, culture duration, and reporting standards; these parameters should be interpreted as context-dependent design variables rather than universal thresholds.

In summary, vascularization is a prerequisite for scale, survival, maturation, and clinical translation of bone organoids (Novosel et al., 2011; Takebe et al., 2013; Auger et al., 2013). Static hydrogels and uncontrolled co-delivery strategies cannot reproduce the ordered angiogenic–osteogenic cascade (Kusumbe et al., 2014; Richardson et al., 2001). Spatiotemporally programmable hydrogels address this gap by coordinating material degradation, mechanical relaxation, biochemical signaling, and cellular remodeling over time (Chaudhuri et al., 2016; Auger et al., 2013), providing a theoretical foundation for engineering vascularized bone organoids that can survive, integrate, and function after implantation (Grigo et al., 2019).

## 3 Dynamic hydrogels: design principles, classification, and comparison with static analogues

Dynamic hydrogels are hydrogel systems capable of time-dependent or cell-responsive changes in mechanical properties, network architecture, ligand availability, or degradation behavior through reversible bonding, degradable linkages, cell-mediated remodeling, or externally applied stimuli (Chaudhuri et al., 2020; Skylar-Scott et al., 2019; Madl and Heilshorn, 2018; Zhou et al., 2025; Kwan et al., 2025). These features may regulate cell aggregation, migration, angiogenesis, osteogenesis, and extracellular matrix remodeling in a spatially and temporally tunable manner (Kusumbe et al., 2014; Ramasamy et al., 2014; Chaudhuri et al., 2020), but the extent depends on hydrogel chemistry, cell composition, culture model, and degree of direct validation in bone organoids. Findings from MSC cultures or related systems should be interpreted as mechanistic or design evidence rather than direct evidence of bone organoid maturation. By integrating materials science with developmental biology, dynamic hydrogels provide a versatile platform for engineering physiologically relevant bone organoids and addressing key challenges such as vascularization, matrix remodeling, osteogenic maturation, and mineral deposition (Ng et al., 2020; Brassard et al., 2021).

### 3.1 Design principles of dynamic hydrogels

Dynamic matrices should support early cell self-organization and vascular invasion while subsequently providing appropriate mechanical stability and biochemical cues for osteogenic maturation and mineralized matrix formation. Four design principles are especially relevant: mechanically permissive remodeling, temporally programmed degradation and factor presentation, stimuli-responsive regulation, and spatial organization of biochemical cues (Burdis and Kelly, 2021; Falcucci et al., 2023).

#### 3.1.1 Tunable network mechanics

Viscoelastic properties, including elastic stiffness and time-dependent stress relaxation, critically regulate cell spreading, migration, cytoskeletal organization, and lineage commitment (Chaudhuri et al., 2016; Mammoto and Ingber, 2010; Zhang H. et al., 2025; Cameron et al., 2011). Compared with predominantly elastic hydrogels, viscoelastic networks allow cells to dissipate local stresses and remodel their surrounding matrix, which is particularly important during early bone organoid formation when mesenchymal stromal cells, endothelial cells, and supporting populations require a mechanically permissive matrix for aggregation, migration, and vascular network assembly (Caliari and Burdick, 2016; Liu et al., 2024).

The matrix should exhibit stage-dependent mechanical behavior: early rapid or moderate stress relaxation can facilitate cell-mediated remodeling and endothelial sprouting; later increased matrix stability supports osteogenic maturation, extracellular matrix deposition, and mineralization (Feng et al., 2025; Yang et al., 2021; Tao et al., 2026). This can be achieved by combining reversible crosslinks with secondary stiffening mechanisms such as photo-crosslinking, ionic reinforcement, enzymatic crosslinking, or mineral incorporation (Guvendiren and Burdick, 2012). The key design goal is not to maximize softness or stress relaxation, but to balance early mechanical permissiveness with long-term structural stability.

#### 3.1.2 Programmed degradation and matrix remodeling

Controlled degradation is central to dynamic hydrogel design. Degradation creates physical space for cell migration, vascular invasion, extracellular matrix deposition, and mineralized tissue formation, and also regulates the relative timing and magnitude of bioactive cues. Rapidly degrading domains may preferentially release pro-angiogenic cues such as VEGF or DMOG during early organoid development, while slowly degrading regions provide sustained presentation of osteoinductive molecules such as BMP-2, together with osteoconductive components such as nano-hydroxyapatite or calcium phosphate particles (Kempen et al., 2009; Patel et al., 2008; Wang et al., 2017; LeGero, 2008). This design should be understood as temporally biased and overlapping rather than strictly sequential.

Degradation kinetics must be matched to the rate of tissue formation. Excessively rapid degradation may compromise mechanical integrity and lead to premature loss of morphogen gradients; overly slow degradation may restrict cell migration, vascularization, and endogenous matrix deposition. Optimal degradation design requires simultaneous consideration of factor release, matrix remodeling, mechanical stability, and tissue maturation.

#### 3.1.3 Stimuli-responsive regulation

Stimuli-responsive hydrogels can respond to external stimuli (light, ultrasound, magnetic fields, electrical stimulation, temperature) or local microenvironmental cues (pH, enzymatic activity, reactive oxygen species, hypoxia) (Rosales and Anseth, 2016; Kloxin et al., 2009; Lutolf et al., 2003; DeForest and Anseth, 2011; Teixeira do Nascimento et al., 2025; Yeingst et al., 2022; Lin et al., 2024; Calderon Moreno et al., 2025). These responses can trigger changes in stiffness, porosity, ligand availability, degradation rate, or bioactive molecule release (Yeingst et al., 2022; Lin et al., 2024). For bone organoid engineering, externally triggered responsiveness allows intervention during specific developmental windows. Light-responsive systems provide high spatial precision for local matrix softening, stiffening, or ligand activation, although penetration depth may be limited in larger or more mineralized constructs (Wei et al., 2017; He et al., 2021). Ultrasound-responsive systems offer deeper tissue penetration and may modulate drug release, enhance mass transport, or activate mechanosensitive and piezoelectric components non-invasively. However, stimulus responsiveness should consider potential cytotoxicity, photothermal effects, acoustic cavitation, off-target degradation, and compatibility with long-term culture; it is a controllable regulatory tool rather than a universally superior design feature.

#### 3.1.4 Spatial patterning of biochemical cues

Bone organoid formation requires spatial organization of biochemical and biophysical signals, as native skeletal development is governed by gradients of morphogens, oxygen tension, matrix stiffness, mineral content, and vascular-derived cues (Kirschner and Anseth, 2013). Spatial control can be achieved through photochemical ligand regulation, localized immobilization or release of bioactive factors, microfluidic patterning, bioprinting, and compartmentalized architectures. Photolabile protecting groups and orthogonal photochemical reactions can regulate ligand presentation, hydrogel degradation, and local release of bioactive signals (Kloxin et al., 2009; DeForest and Anseth, 2011). Microfluidic patterning, 3D bioprinting, sacrificial templating, gradient crosslinking, and compartmentalized architectures can generate spatially organized cues (Kolesky et al., 2014; Kolesky et al., 2016; Miller et al., 2012; Bhatia and Ingber, 2014; He et al., 2021; Kirschner and Anseth, 2013; Jeon et al., 2011; Kumar et al., 2026), but architectural patterning alone does not demonstrate developmental self-organization. The degree of spatial precision depends strongly on the patterning method, construct size, optical or acoustic penetration, diffusion of soluble factors, and long-term stability of immobilized cues; claims of “high precision” should be made cautiously for large, dense, or mineralized constructs.

### 3.2 Classification of dynamic hydrogels

Dynamic hydrogels can be classified by their dominant mechanism, although many systems combine multiple dynamic mechanisms within a single platform (Wojtecki et al., 2011; Webber et al., 2016; McKinnon et al., 2014; Rod et al., 2013; Hall and Miyake, 2000).

#### 3.2.1 Viscoelastic and stress-relaxing hydrogels

Viscoelastic or stress-relaxing hydrogels dissipate applied stress over time through reversible network rearrangement. Chaudhuri et al. (2016) developed alginate hydrogels with independently tunable stiffness and stress-relaxation kinetics and showed that faster stress relaxation promoted mesenchymal stem-cell spreading, proliferation, osteogenic differentiation, matrix deposition, and bone formation *in vivo*. Substrate stress relaxation directly regulates cell spreading by permitting cell-mediated matrix deformation (Chaudhuri et al., 2015). Time-dependent mechanics can be introduced through dynamic covalent bonds or supramolecular interactions, including hydrazone, disulfide, boronate-ester, host–guest, hydrogen-bonding, ionic, metal–ligand, and DNA hybridization mechanisms (Rosales and Anseth, 2016; Chaudhuri et al., 2020; Webber et al., 2016; McKinnon et al., 2014; Rod et al., 2013; Hall and Miyake, 2000; Lloyd et al., 2023; Rowan et al., 2002; Khetan et al., 2013). McKinnon et al. (2014) created reversible hydrazone-crosslinked PEG hydrogels as cytocompatible viscoelastic 3D culture systems; guest–host hyaluronic acid hydrogels exhibit shear-thinning, self-healing, and tunable mechanical properties (Rod et al., 2013). These dynamic motifs allow network remodeling under cell-generated forces, promoting cell spreading, migration, proliferation, aggregation, and matrix deposition.

In bone organoid engineering, such behavior may support early mesenchymal condensation, a critical process in skeletal development (Hall and Miyake, 2000; Hall and Miyake, 1992), and later osteogenic matrix remodeling. However, excessively fast relaxation may compromise long-term architecture; overly slow relaxation may restrict cell-mediated remodeling. Both relaxation timescale and initial elastic modulus should be optimized according to cell type, organoid size, culture duration, and stage of osteogenic maturation.

#### 3.2.2 Degradable and degradation-programmed hydrogels

Degradable dynamic hydrogels undergo hydrolytic, enzymatic, oxidative, or cell-mediated cleavage of network linkages. Lutolf et al. (2003) developed synthetic MMP-sensitive PEG hydrogels with protease-cleavable peptide crosslinkers that permit cell-mediated invasion and matrix remodeling. Khetan et al. (2013) showed that degradation-mediated cellular traction regulates human MSC spreading and differentiation in 3D matrices. Patterson and Hubbell (2010) demonstrated that the sequence and accessibility of protease-sensitive peptide crosslinkers can tune enzymatic degradation of PEG hydrogels and cell invasion. Photodegradable systems provide another form of degradation programming; Kloxin et al. (2009) created photodegradable PEG hydrogels whose light exposure dynamically alters hydrogel mechanics and structure *in situ*, and DeForest and Anseth (2011) developed click-based hydrogels with orthogonal photoconjugation and photocleavage reactions.

In bone organoid engineering, degradation-programmed hydrogels may coordinate matrix remodeling with tissue formation, but direct applications remain limited. Available evidence derives mainly from MSC culture and related tissue-engineering models, where controlled matrix degradation has been associated with cell spreading, lineage specification, vascular invasion, and matrix deposition (Lutolf et al., 2003; Peppas et al., 2000; Siepmann and Siepmann, 2012). By incorporating domains with different degradation rates, these hydrogels can support staged release of pro-angiogenic, osteogenic, or immunomodulatory cues. Richardson et al. (2001) demonstrated that distinct release profiles provide temporal control over angiogenic signaling; Kempen et al. (2009) showed that sequential local delivery of VEGF and BMP-2 improved vascularized bone formation. These studies support temporally biased factor presentation but do not establish that complete degradation-programmed systems have been validated in bone organoids. Core–shell, multilayered, or compartmentalized architectures can further enable spatially and temporally biased presentation (Kempen et al., 2009; Richardson et al., 2001). Degradation-mediated release is influenced by diffusion, local enzyme concentration, cell density, pH, and matrix composition; such systems should be described as tunable rather than perfectly precise (Siepmann and Siepmann, 2012; Lin and Anseth, 2009).

#### 3.2.3 Stimuli-responsive hydrogels

Photoresponsive hydrogels enable spatiotemporal regulation of matrix degradation, stiffness, and ligand presentation. Kloxin et al. (2009) and DeForest and Anseth (2011) provided useful design principles, although light penetration, phototoxicity, and photoinitiator effects require careful consideration. Ultrasound-responsive hydrogels offer non-invasive actuation in deeper constructs; Huebsch et al. (2014) reported reversibly crosslinked hydrogels that could be disrupted by ultrasound and self-heal, but ultrasound parameters must be optimized to avoid excessive heating, cavitation, or matrix disruption (ter Haar, 2007). Microenvironment-responsive hydrogels can use pH-sensitive or ROS-sensitive linkages (Koetting et al., 2015; Broaders et al., 2011), but pH and ROS levels vary substantially with cell type, culture duration, oxygen tension, and inflammatory status, so these systems are tunable rather than perfectly precise. Electroactive and piezoelectric hydrogel composites may provide osteogenesis-relevant electrical or electromechanical cues (Hronik-Tupaj et al., 2011; Damaraju et al., 2017; Rajabi et al., 2015; Tandon et al., 2018), but filler concentration, stimulation intensity, exposure duration, and long-term cytocompatibility must be evaluated, as nanoscale fillers may induce oxidative stress or cytotoxicity (Nel et al., 2006).

#### 3.2.4 Dynamic double-network and multi-network hydrogels

Double-network and multi-network hydrogels contain two or more interpenetrating or mutually crosslinked networks. Classical double-network hydrogels were first demonstrated by Gong et al. (2003), but not all double-network hydrogels are dynamic; they are dynamic only when at least one network contains reversible bonds, degradable linkages, stimuli-responsive motifs, or cell-remodelable components. In dynamic double-network systems, one network may dissipate energy or permit stress relaxation, while another maintains structural integrity. Gelatin methacryloyl (GelMA) is commonly used as a photocrosslinkable and cell-adhesive network (Nichol et al., 2010; Yue et al., 2015); GelMA/alginate systems combine cell adhesiveness with rapid ionic crosslinking and reversible interactions. Jia et al. (2016) developed a GelMA/alginate/PEGTA blended bioink for 3D bioprinting of perfusable vascular constructs. DNA-based hydrogels provide reversible and sequence-programmable interactions (Um et al., 2006; Xing et al., 2011; Cangialosi et al., 2017). For bone organoid engineering, dynamic multi-network hydrogels may balance early matrix adaptability with long-term mechanical stability. Increasing network complexity may complicate reproducibility, diffusion, degradation kinetics, and interpretation of cell–matrix interactions; multi-network design should be guided by clearly defined biological objectives rather than mechanical reinforcement alone.

## 4 Spatiotemporal design strategies: demonstrated principles and testable hypotheses

The strategies in this section differ in their level of validation. Some are supported by direct evidence from bone organoid or organoid-like models, while others are inferred from related bone-regeneration, MSC, vascular, cartilage, wound-healing, or general hydrogel studies. Table 1 summarizes the evidence status of each strategy.

**TABLE 1:** Evidence classification of spatiotemporal biomaterial strategies for angiogenic–osteogenic coupling in bone organoids and related models.

Strategy	Core design	Representative biomaterials	Bioactive cues/stimulation	Evidence status	Main rationale and limitation	Representative references
Degradation-mediated temporally biased release	Differential degradation/remodeling generates an early angiogenic bias with more sustained osteogenic signaling	GelMA, alginate, HYA, silk fibroin, calcium phosphate, nHAp-containing hydrogels	VEGF, DMOG, BMP-2	Indirect evidence; proposed strategy	Temporally controlled delivery can coordinate vascular and osteogenic responses, but the complete proposed configuration has not been directly validated in bone organoids and release remains dependent on diffusion, degradation and cell-mediated remodeling	Kempen et al. (2009), Wang et al. (2007), Schipani et al. (2009), Elvidge et al. (2006), Richardson et al. (2001), LeGeros (2008), Siepmann and Siepmann (2012), Lin and Anseth (2009), Urist (1965), Wozney et al. (1988), Lee and Mooney (2012), Burdick and Prestwich (2011), Kundu et al. (2013), Freiberg and Zhu (2004), Murphy and Atala (2014), Branco et al. (2010), Lindsey et al. (2015), Pablos et al. (2024)
Viscoelastic modulation	Stress relaxation permits cell spreading, traction generation and matrix remodeling	Alginate, HYA–collagen and dynamically crosslinked hydrogels	Time-dependent mechanical cues	Indirect evidence	Faster relaxation can promote MSC spreading and osteogenic differentiation and may support vascular organization; however, effects depend on initial modulus, ligand presentation, cell type and culture stage	Chaudhuri et al. (2016), Chaudhuri et al. (2020), Wei et al. (2023), Lou et al. (2018)
Dynamic mechanical/piezoelectric stimulation	External mechanical or ultrasound stimulation generates mechanical or electromechanical signals	PVDF-, barium-titanate- and calcium-phosphate-based piezoelectric materials	Ultrasound, electrical/electromechanical stimulation	Indirect evidence; experimental hypothesis	Primary studies support effects on MSC osteogenic differentiation, but evidence for endothelial responses and coordinated angiogenic–osteogenic maturation in bone organoids remains limited; outcomes are highly parameter-dependent	Hronik-Tupaj et al. (2011), Damaraju et al. (2017), Rajabi et al. (2015), Tandon et al. (2018), Fukada and Yasuda (1957), Bassett and Becker (1962), Heckman et al. (1994), Kristianse et al. (1997), TRUST Investigators writing group et al. (2016)
Multicellular spatial organization	Spatial arrangement and co-culture of endothelial, mesenchymal and osteogenic cells using bioprinting, microfluidics or compartmentalized assembly	PEG-based, GelMA and other cell-compatible hydrogels	Cell–cell and paracrine signaling	Indirect evidence from related multicellular models	Spatial organization can support vascular stabilization and osteogenic–vascular interactions, but pattern fidelity does not necessarily indicate functional perfusion or mature bone-organoid formation	Au et al. (2008), Kolesky et al. (2014), Miller et al. (2012), Kumar et al. (2026), Koike et al. (2004), Miller et al. (2010), Hanjaya-Putra et al. (2010), Truong et al. (2026), Byambaa et al. (2017)

Abbreviations: BMP-2, bone morphogenetic protein 2; DMOG, dimethyloxalylglycine; GelMA, gelatin methacryloyl; HYA, hyaluronic acid; MSC, mesenchymal stromal/stem cell; nHAp, nano-hydroxyapatite; PEG, poly (ethylene glycol); PVDF, poly (vinylidene fluoride); VEGF, vascular endothelial growth factor.

### 4.1 Degradation-mediated sequential release

This proposed strategy leverages differential degradation kinetics across hydrogel compartments to create temporally biased presentation of angiogenic and osteogenic cues. Fast-degrading regions may release pro-angiogenic signals during an early phase, while slow-degrading compartments provide more sustained osteoinductive cues. The complete bilayer hydrogel has not been directly demonstrated in a bone organoid model. Richardson et al. (2001) showed that temporally controlled growth factor release modulates tissue formation; Kempen et al. (2009) showed that sequential delivery of VEGF and BMP-2 benefits bone regeneration. VEGF or hypoxia-mimetic agents such as DMOG could be considered as candidate early-phase cues because HIF–VEGF signaling can promote angiogenic–osteogenic coupling in skeletal and tissue-engineering models (Wang et al., 2007; Schipani et al., 2009; Elvidge et al., 2006); slow-degrading phases deliver BMP-2, calcium phosphate, or nano-hydroxyapatite for prolonged osteoinductive and osteoconductive stimulation (LeGero, 2008; Urist, 1965; Wozney et al., 1988). This design can be realized with degradable polymers including GelMA, alginate, hyaluronic acid, and silk fibroin, often paired with factor-loaded microspheres, and is compatible with emulsion fabrication, microfluidics, and extrusion bioprinting (Nichol et al., 2010; Yue et al., 2015; Lee and Mooney, 2012; Burdick and Prestwich, 2011; Kundu et al., 2013; Freiberg and Zhu, 2004; Murphy and Atala, 2014). Actual release kinetics are governed by polymer concentration, crosslinking density, enzymatic sensitivity, mesh size, and local cellular activity rather than nominal composition alone (Siepmann and Siepmann, 2012; Lin and Anseth, 2009; Branco et al., 2010; Lindsey et al., 2015; Pablos et al., 2024); release sequences must be experimentally validated under target conditions.

### 4.2 Viscoelastic modulation of cell behavior

Matrix viscoelasticity is characterized by stress relaxation, the gradual decay of stress under constant deformation. Native ECMs and hydrogel mimics are viscoelastic, and cells respond to both stiffness and time-dependent matrix remodeling (Chaudhuri et al., 2020). Lou et al. (2018) showed that stress-relaxing hyaluronic acid (HYA)–collagen hydrogels promote cell spreading and focal adhesion formation; Chaudhuri et al. (2016) showed that faster stress relaxation in alginate hydrogels enhances MSC spreading, osteogenic differentiation, and matrix deposition. For bone organoid engineering, faster relaxation may facilitate endothelial protrusion, migration, and network assembly when paired with appropriate biochemical cues (Wei et al., 2023), but this conclusion is currently supported mainly by related hydrogel and cell-culture studies. The effect of viscoelasticity on lineage specification is context-dependent: fast relaxation generally enhances osteogenesis, while more stable matrices may favor chondrogenic phenotypes (Chaudhuri et al., 2016). This stage-dependent requirement aligns with endochondral ossification, where an early cartilage template needs structural stability and subsequent vascular invasion and bone formation benefit from greater matrix permissiveness.

### 4.3 Dynamic mechanical stimulation and piezoelectric hydrogels

Mechanical forces regulate bone development and homeostasis (Leslie-Barbick et al., 2011; Bonewald and Johnson, 2008). Coupling ultrasound stimulation with piezoelectric hydrogels delivers localized electromechanical cues inspired by bone’s intrinsic piezoelectricity (Rajabi et al., 2015; Tandon et al., 2018). Piezoelectric fillers such as barium titanate, PVDF, and some calcium-phosphate-based materials can convert mechanical deformation into transient electrical signals (Rajabi et al., 2015; Tandon et al., 2018; Fukada and Yasuda, 1957; Bassett and Becker, 1962). Studies in MSC-based systems suggest that electrical or electromechanical stimulation may influence osteogenic differentiation (Hronik-Tupaj et al., 2011; Damaraju et al., 2017), but evidence for endothelial effects, coordinated vascular–osteogenic maturation, and bone-organoid formation remains limited. In principle, time-dependent mechanical stimulation could provide different physical cues during vascular organization and osteogenic maturation, but this remains a design hypothesis. Evidence from fracture-healing studies is mixed: early trials reported beneficial effects of low-intensity pulsed ultrasound (Heckman et al., 1994; Kristianse et al., 1997), whereas the larger TRUST randomized trial did not demonstrate significant improvement (TRUST Investigators writing group et al., 2016). The effects of ultrasound depend on frequency, intensity, duty cycle, exposure duration, construct geometry, acoustic attenuation, local heating, cavitation, and piezoelectric fillers. Studies should include sham stimulation, thermal monitoring, and independent assessment of vascular and osteogenic outcomes. Ultrasound-responsive or piezoelectric hydrogels should be regarded as experimental platforms rather than established solutions for angiogenic–osteogenic coupling.

### 4.4 Multi-cellular loading and spatial organization

Bone organoid-like constructs rely on coordinated interactions among endothelial cells, mesenchymal stromal/stem cells, osteogenic progenitors, and sometimes perivascular or immune cells. Stromal support is critical for stable vascular structures; Koike et al. (2004) showed that mesenchymal precursors enable long-lasting functional vessels, and Au et al. (2008) found that bone marrow MSCs provide perivascular-like support for durable vasculature. Co-encapsulation of endothelial and osteogenic cells in a permissive hydrogel is therefore a foundational strategy. Hydrogel degradability, adhesive ligands, stiffness, and viscoelasticity collectively regulate cell spreading, vascular morphogenesis, and stem cell fate (Lutolf and Hubbell, 2005; Chaudhuri et al., 2016; Peppas et al., 2000). For example, PEG–peptide hydrogels (Miller et al., 2010) and substrate-mechanics-mediated endothelial tubulogenesis (Hanjaya-Putra et al., 2010) illustrate how matrix chemistry and mechanical properties govern vascular assembly. Spatial patterning via 3D bioprinting, microfluidics, sacrificial templating, or compartmentalized assembly further enhances structural organization (Kolesky et al., 2014; Miller et al., 2012; Truong et al., 2026). Byambaa et al. demonstrated that bioprinted osteogenic and vasculogenic patterns support both vascular network formation and osteogenic differentiation (Byambaa et al., 2017), though these systems do not fully recapitulate native bone hierarchy. Multicellular spatial organization is promising but increases system complexity; evaluation must cover long-term vascular stability, perfusability, and matrix mineralization, not just initial pattern fidelity. For 3D-printed or bioprinted constructs, the trade-off between printability, self-healing capacity, and long-term structural stability represents a key design challenge (Kumar et al., 2026).

## 5 Experimentally demonstrated and proposed dynamic hydrogel strategies for bone organoids and related systems

Dynamic hydrogel strategies for bone organoid engineering remain at an early stage. The GelMA/DNA dual-network hydrogel has been directly evaluated in a woven bone-like organoid model, whereas the bilayer degradation-mediated hydrogel, HYA-Tyr hydrogel, and microsphere-assembled hydrogel are supported mainly by related studies. Each system is classified as direct evidence, indirect evidence, or a proposed design concept. Table 2 summarizes the composition, dynamic features, biological rationale, intended model, and evidence status.

**TABLE 2:** Evidence classification of dynamic hydrogel strategies for bone organoids and related bone tissue-engineering systems.

Hydrogel system	Main composition	Dynamic feature	Model or intended application	Evidence status	Main biological rationale or outcome	Main limitation
GelMA/DNA dual-network hydrogel	GelMA and DNA-based supramolecular network	Reversible DNA interactions; enhanced viscoelasticity and stress relaxation	Woven bone-like organoid	Direct evidence	Supports BMSC spreading, aggregation, osteogenic differentiation, collagen deposition, and mineralization	Vascularization, long-term maturation, hierarchical remodeling, and mechanical competence require further evaluation
Bilayer degradation-mediated hydrogel	Proposed fast-remodeling angiogenic compartment and slow-remodeling osteogenic compartment, such as GelMA- and silk-fibroin-based layers	Differential degradation and temporally biased factor release	Proposed vascularized bone organoid or related bone-regeneration construct	Proposed design; indirect evidence	May provide an early angiogenic bias followed by relatively sustained osteogenic and mineralization-related cues	The complete bilayer system has not been directly demonstrated in a bone organoid model; release depends on degradation, diffusion, and cellular remodeling
Viscoelastic HYA-Tyr hydrogel	HYA-Tyr	Tunable viscoelasticity and stress relaxation	Proposed cartilage-related or endochondral bone organoid model	Indirect evidence; proposed application	May support cell-mediated remodeling, chondrogenesis, cartilage-like matrix formation, and mechanics–metabolism interactions	Direct evidence linking HYA-Tyr hydrogels with hypoxia, histone lactylation, chondrogenesis, and subsequent bone formation remains limited
Microsphere-assembled hydrogel	Annealed or interlinked microspheres based on PEG, GelMA, HYA, or related polymers	Modular assembly, injectability, and intrinsic microporosity	Proposed callus-like or injectable bone organoid construct	Indirect evidence; proposed application	Microporosity and modularity may facilitate cell migration, tissue infiltration, vascular ingrowth, and spatial organization of osteogenic or chondrogenic niches	Direct validation in bone organoids is lacking; current evidence is mainly from wound-healing, callus, and related tissue-engineering models

Direct evidence refers to experimental evaluation of the complete hydrogel system in a bone organoid or organoid-like bone model. Indirect evidence refers to findings obtained from related MSC, cultures, bone-regeneration models, cartilage or endochondral ossification models, wound-healing studies, or general biomaterial systems. Proposed design refers to a strategy formulated from related studies that has not yet been directly validated as a complete bone organoid hydrogel system. BMSC, bone marrow-derived mesenchymal stromal/stem cell; GelMA, gelatin methacryloyl; HYA-Tyr/HYA, hyaluronic acid;PEG, poly (ethylene glycol); DNA, deoxyribonucleic acid.

### 5.1 GelMA/DNA dual-network hydrogels for woven bone-like organoids—direct evidence

Woven bone is an immature matrix formed during embryonic skeletogenesis and early fracture repair, characterized by rapid deposition and relatively disorganized collagen structure (Marsell and Einhorn, 2011; Shapiro, 2008). GelMA is widely used because of its photocrosslinkability, tunable mechanics, and cell-adhesive motifs (Nichol et al., 2010; Yue et al., 2015), but conventional covalently crosslinked GelMA has limited capacity for dynamic remodeling after gelation. Integrating GelMA with dynamic supramolecular DNA networks addresses this limitation; DNA hydrogels assemble through programmable base-pairing and exhibit reversible, stimulus-responsive interactions (Um et al., 2006; Xing et al., 2011; Cangialosi et al., 2017; Shi R. et al., 2025). These reversible interactions may increase stress relaxation and viscoelastic remodeling compared with purely covalent GelMA networks (Chaudhuri et al., 2016; Chaudhuri et al., 2015).

The GelMA/DNA dual-network hydrogel is the clearest example of a dynamic hydrogel directly evaluated in a woven bone-like organoid model (Zhu et al., 2025). The dynamic network supported spreading, migration, and aggregation of bone-marrow-derived mesenchymal stromal/stem cells and contributed to woven bone-like features, including disorganized collagen, osteocyte-like cells, and mineralized matrix (Zhu et al., 2025). The construct also showed pro-osteogenic and osseointegrative effects in a calvarial defect model (Zhu et al., 2025). However, the construct is more accurately described as a woven bone-like organoid or organoid-like bone construct rather than a fully mature bone equivalent; its vascularization, marrow-like organization, hierarchical remodeling, mechanical competence, long-term maturation, and functional integration require further evaluation. This study provides direct evidence for the use of a dynamic hydrogel in a bone organoid-like model but does not establish general applicability across all bone organoid systems.

### 5.2 Bilayer degradation-mediated hydrogel for temporally biased angiogenic–osteogenic coupling—proposed design concept

The bilayer configuration is a testable design hypothesis formulated from independent findings on growth-factor delivery, hydrogel degradation, and vascularized bone regeneration, not a published complete system. A hypothetical bilayer construct could contain two spatially defined compartments with different remodeling and release profiles: one providing stronger early angiogenic support, the second providing more sustained osteogenic or mineralization-related cues. The purpose is not to impose a rigid sequence but to establish an early angiogenic bias while allowing osteogenic differentiation and matrix deposition to proceed concurrently. The rationale is informed by studies showing that temporally controlled VEGF delivery can regulate angiogenic responses and sequential VEGF/BMP-2 delivery can improve bone regeneration (Kempen et al., 2009; Patel et al., 2008; Richardson et al., 2001; Silva and Mooney, 2007). GelMA and silk fibroin provide examples of materials with distinct crosslinking and degradation characteristics (Yue et al., 2015; Kundu et al., 2013; Wang et al., 2008). Essential measurements would include degradation half-life of each layer, residual modulus, mesh size, layer thickness, factor loading, burst release, release duration, spatial factor distribution, endothelial-network formation, osteogenic differentiation, mineral deposition, organoid viability, and host anastomosis after implantation. Direct comparison with single-layer and simultaneous-release controls is necessary.

### 5.3 Viscoelastic HYA-Tyr hydrogels for cartilage-related and endochondral bone organoid models—indirect evidence and proposed application

HYA-Tyr hydrogels are a candidate material platform, not an experimentally established bone-organoid hydrogel system. HYA-Tyr refers to hyaluronic acid chemically modified with tyramine or phenol groups and enzymatically crosslinked (Darr and Calabro, 2009). The proposed rationale is that HYA-Tyr hydrogels may provide a hydrated, cell-interactive, and mechanically adaptable environment for cartilage-related or endochondral models. Related studies show that stress-relaxing matrices can influence cell spreading, matrix remodeling, and mechanotransduction (Chaudhuri et al., 2016; Chaudhuri et al., 2015; Lou et al., 2018); hypoxia-responsive signaling can regulate chondrocyte survival and SOX9-associated differentiation (Schipani et al., 2001); histone lactylation has been identified as a mechanism linking cellular metabolism to gene regulation (Zhang et al., 2019). However, these observations do not establish a continuous causal pathway from HYA-Tyr hydrogel mechanics to oxygen concentration, glycolysis, lactate accumulation, histone lactylation, chondrogenesis, and subsequent endochondral bone formation. A rigorous test would require quantitative measurement of oxygen concentration, HIF-1α stabilization, lactate production, histone lactylation, SOX9, COL2A1, and ACAN expression, cartilage-matrix deposition, vascular invasion, and subsequent mineralization, plus mechanistic experiments with inhibition or genetic perturbation of lactate production and histone lactylation.

### 5.4 Injectable microsphere-assembled hydrogels for callus-like or injectable bone organoid constructs—indirect evidence and proposed application

Microsphere-based hydrogels provide modularity, injectability, and microporosity, whereas callus-like organoids aim to reproduce the multicellular and developmental organization of fracture repair (Einhorn, 1998; Einhorn and Gerstenfeld, 2015; Bahney et al., 2019). Evidence that a microporous scaffold promotes cell infiltration or wound healing should not be interpreted as evidence that it forms a callus-like bone organoid. Microporous annealed particle (MAP) hydrogels assembled from microgel building blocks can promote cell infiltration and accelerate wound healing *in vivo* (Griffin et al., 2015), providing indirect evidence that microporosity may facilitate cell migration and tissue infiltration. However, this does not demonstrate formation, vascularization, or maturation of a callus-like bone organoid. A relevant callus-organoid study reported developmentally engineered callus organoid bioassemblies with predictive effects in a long-bone healing model (Nilsson et al., 2020), closer to the organoid concept but still with undefined material dependence, vascularization, osteoclast contribution, immune regulation, and long-term remodeling. Microspheres may also function as independent micro-niches loaded with osteogenic cells, chondrogenic progenitors, endothelial cells, calcium phosphate, or controlled-release morphogens; PEG microsphere-based modular scaffolds support cell encapsulation and macroporous structure formation. Nevertheless, the ability of these systems to support self-organizing bone organoids, stable vascular networks, endochondral progression, or long-term mineralized maturation has not been directly established. Microsphere-assembled hydrogels should be classified as indirect evidence with a proposed application. Future studies should evaluate microsphere composition, pore size, interparticle bonding, degradation kinetics, factor-loading efficiency, release duration, vascular network formation, chondrogenesis, mineralization, and organoid maturation in a defined multicellular model.

### 5.5 Evidence status and implications

The systems discussed should not be regarded as equivalent examples of established dynamic hydrogels for bone organoids. The GelMA/DNA dual-network hydrogel provides the clearest direct evidence in a woven bone-like organoid model (Zhu et al., 2025). Callus-like organoid assemblies provide related organoid-level evidence but are not necessarily equivalent to dynamic hydrogel systems. The bilayer design and HYA-Tyr design are proposed or hypothesis-generating frameworks; MAP and other microsphere-assembled hydrogels are adjacent biomaterial platforms with potential but unproven relevance to bone organoids. Direct validation should include quantitative assessment of cellular self-organization, vascularization, endothelial stability, osteogenic differentiation, extracellular matrix deposition, mineralization, organoid maturation, and functional integration.

## 6 Relating hydrogel parameters to biological outcomes: current evidence and quantitative limitations

The biological performance of hydrogel-based bone-organoid constructs is determined by the combined effects of degradation behavior, viscoelasticity, bioactive cue presentation, and mechanical support. Establishing quantitative structure–function relationships is essential for rational design. However, direct numerical comparison among studies remains difficult because hydrogel composition, cell source, organoid size, culture medium, loading conditions, measurement methods, and observation time vary substantially. In addition, important parameters such as mesh size, stress-relaxation timescale, degradation half-life, growth-factor dose, and release duration are not consistently reported. To facilitate standardized reporting and cross-study comparison, Table 3 proposes a quantitative reporting framework that links each key hydrogel parameter to its potential biological relevance, identifies current sources of heterogeneity, and specifies recommended reporting items. To provide a quantitative overview of the available evidence, Table 4 summarizes material parameters and biological outcomes reported in representative bone-organoid, organoid-like, and related hydrogel studies. The reported values should be interpreted as context-dependent design observations rather than universal thresholds. The relationships below should be interpreted as design trends rather than universal quantitative thresholds. Figure 2 provides a conceptual overview of these relationships, illustrating how degradation, stress relaxation, release kinetics, and mechanical strength interact to influence vascularization, matrix remodeling, and osteogenic maturation.

**TABLE 3:** Quantitative reporting framework for dynamic hydrogel design in bone-organoid engineering.

Parameter	Potential biological relevance	Current reporting status	Main source of heterogeneity	Recommended reporting items
Initial elastic or storage modulus	Influences cell spreading, traction generation, matrix stability, and tissue organization	Reported inconsistently across studies	Polymer composition, crosslinking method, hydration, testing mode, and strain rate	Modulus, test mode, strain, frequency, temperature, and measurement time
Stress-relaxation timescale	Regulates cell-mediated matrix remodeling and lineage-associated behavior	Often reported, but testing protocols differ	Applied strain, relaxation model, initial modulus, and fitting method	Full relaxation curve, characteristic relaxation time, applied strain, and initial modulus
Degradation timescale	Controls pore formation, cell invasion, factor release, and structural persistence	Frequently measured under nonphysiological conditions	Enzyme concentration, pH, cell density, swelling, and tissue implantation site	Mass loss, residual modulus, enzyme conditions, degradation half-life, and time-dependent porosity
Mesh size or pore size	Influences diffusion, cell migration, vascular invasion, and mineral deposition	Not consistently reported for hydrated or remodeled gels	Imaging method, swelling, degradation, and cell-mediated remodeling	Measurement method, hydrated state, pore-size distribution, and temporal changes
Growth-factor loading and release duration	Determines local signal intensity and exposure time	Highly variable and often difficult to compare	Factor binding, diffusion, degradation, carrier type, and burst release	Total loading, initial burst, cumulative release, release duration, and local concentration
Mechanical reinforcement or mineral content	Supports structural maintenance and load transfer	Reported in related bone-regeneration models but rarely standardized for organoids	Particle type, size, distribution, and hydrogel composition	Mineral fraction, particle size, dispersion, modulus, strength, and cytocompatibility
Biological outcome	Links material parameters to function	Usually reported using different endpoints	Cell source, organoid size, culture duration, and assay methods	Vascular density, branch length, lumen formation, mineral density, gene expression, viability, and host integration

**TABLE 4:** Selected quantitative parameters and biological outcomes reported in representative dynamic hydrogel studies.

Study	Evidence level	Material/model	Quantitative finding	Biological implication
Chaudhuri et al. (2016)	Indirect	RGD (arginine-glycine-aspartate)-alginate hydrogel; 3D D1 MSC culture	Initial modulus: 9 or 17 kPa; stress-relaxation half-time: approximately 1 min–1 h; RGD: 150 or 1,500 μM; negligible degradation over 7 days	Faster stress relaxation promoted MSC spreading and osteogenesis; at approximately 17 kPa, rapidly relaxing gels supported mineralized, collagen-I-rich, interconnected bone-like matrix formation after 14 days
Kloxin et al. (2009)	Indirect	Photodegradable PEG-based hydrogels with encapsulated hMSCs and fibrosarcoma cells	Complete hydrogel degradation occurred within approximately 10 min under 365 nm irradiation at 10 mW/cm^2^. Reducing crosslinking density promoted hMSC spreading, while photorelease of RGDS on day 10 increased GAG production approximately fourfold by day 21	Dynamic control of hydrogel mechanics and ligand presentation regulates cell spreading, migration, viability, and hMSC chondrogenic differentiation
Lutolf et al. (2003)	Indirect	MMP-sensitive PEG hydrogels containing RGD adhesion ligands; human fibroblasts and rat cranial bone-defect model with BMP-2	Fibroblasts invaded >1 mm within 1 week. MMP-sensitive gels enabled complete cell infiltration and bone formation within 4 weeks; bone regeneration used 5 μg BMP-2 per defect	MMP-mediated matrix degradation, together with cell adhesion, enables cell invasion, hydrogel remodeling, and enhanced tissue regeneration
Richardson et al. (2001)	Indirect	Biodegradable PLG scaffold delivering VEGF and PDGF in rat and mouse models	Dual delivery increased vessel density and maturity; 88% of vessels were mature after 4 weeks	VEGF promotes vessel formation, while PDGF promotes vessel maturation; sequential dual-factor delivery supports stable angiogenesis
Kempen et al. (2009)	Indirect	PLGA/PPF scaffold with gelatin hydrogel for sequential VEGF and BMP-2 delivery; tested in ectopic and orthotopic rat bone-defect models	VEGF showed an early burst release (84%–90%), while BMP-2 was released gradually over 56 days. Combined delivery significantly increased ectopic bone formation (p = 0.008) but not orthotopic bone formation (p = 0.10)	Early VEGF release enhances vascularization and supports BMP-2-induced bone formation, but the effect depends on the implantation site and release timing
Zhu et al. (2025)	Direct	Dynamic GelMA/DNA dual-network hydrogel containing catalase, cultured with BMSCs to form woven bone organoids; tested in rat skull defects	After 21 days of culture, the organoids reached a maximum compressive strength of 187 kPa. After 4 weeks *in vivo*, they covered approximately 50% of the skull defect	The viscoelastic hydrogel promotes BMSC migration, self-organization, osteogenic differentiation, and mineralization, leading to woven bone formation and enhanced bone regeneration
Lou et al. (2018)	Indirect	Dynamic HA–collagen type I interpenetrating network (IPN) hydrogels with hydrazone crosslinks; 3D culture of human MSCs	Stress relaxation was tunable: τ_1_/_2_ decreased from ∼2,200 s to ∼560 s as HA concentration decreased from 2% to 1%. Faster-relaxing gels promoted MSC protrusions up to ∼100 μm, collagen-fiber realignment, and focal-adhesion formation	ECM-like viscoelasticity and fibrillar architecture enable cell spreading, matrix remodeling, and mechanosignaling in 3D culture

**FIGURE 2:**
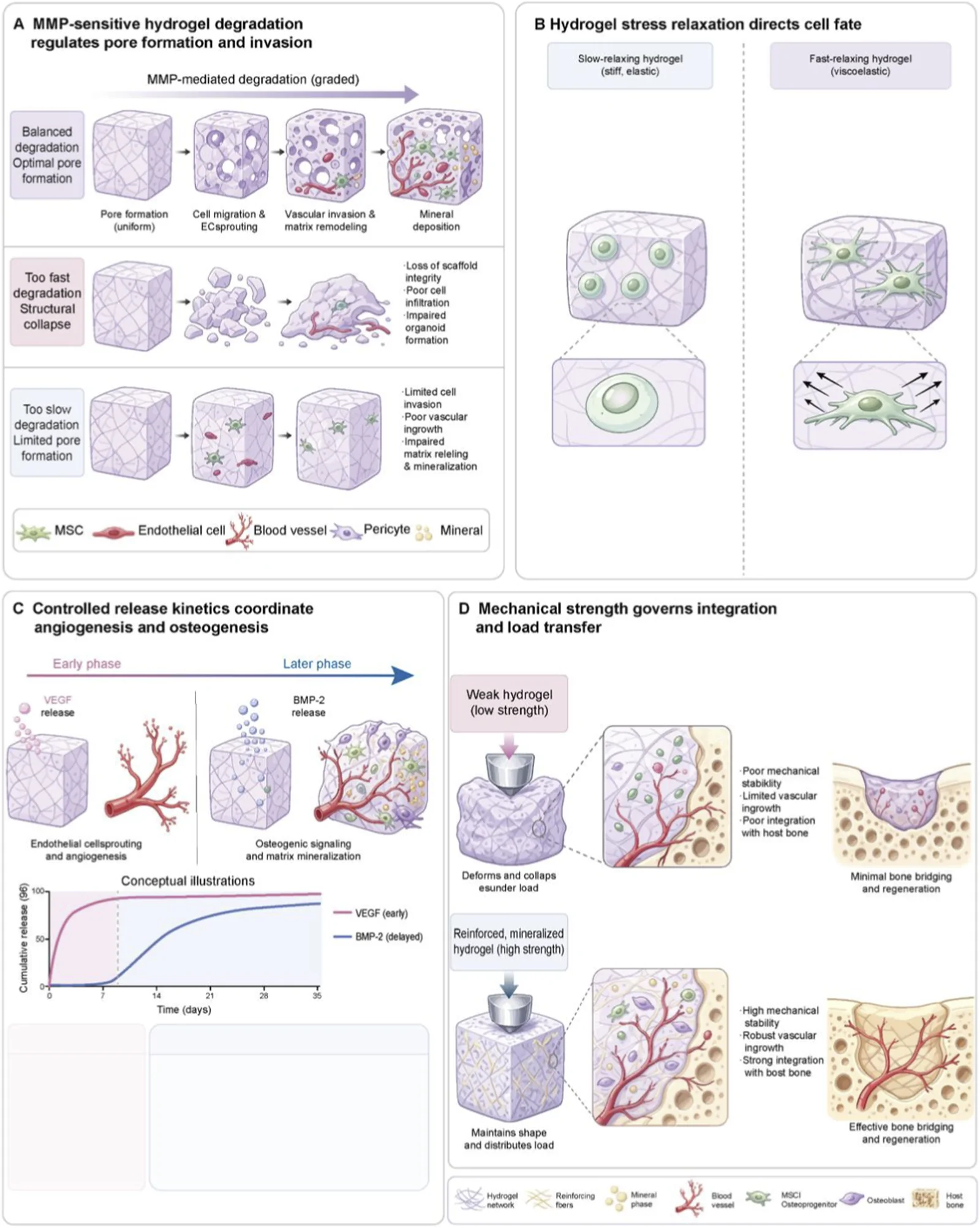
Conceptual relationships between dynamic hydrogel parameters and biological outcomes in vascularized bone organoid engineering. The figure summarizes design hypotheses and trends derived from bone organoid studies and related hydrogel, MSC, cartilage, vascularization, and bone-regeneration models; it is not intended to define universal quantitative thresholds. **(A)** MMP-sensitive hydrogel degradation may regulate pore formation and tissue invasion. A balanced degradation profile may support cell migration, endothelial sprouting, matrix deposition, and subsequent mineralization, whereas excessively rapid or slow degradation may compromise structural stability or restrict cellular invasion. **(B)** Stress relaxation may influence cell morphology, traction generation, and matrix remodeling. The effects on lineage specification depend on initial modulus, ligand presentation, cell type, and culture stage. **(C)** Temporally biased release kinetics may coordinate angiogenic and osteogenic signaling. Early enrichment of VEGF or other pro-angiogenic cues may support endothelial sprouting, whereas delayed or sustained presentation of BMP-2, osteogenic ligands may support osteogenic differentiation and mineralization. The cumulative release curves are conceptual illustrations and are not reproduced from a specific published dataset. Actual release profiles depend on diffusion, swelling, degradation, factor binding, and cell-mediated remodeling. **(D)** Hydrogel mechanical strength may influence structural maintenance, load transfer, vascular ingrowth, and host-tissue integration. Weak matrices may deform or collapse, whereas reinforced or mineral-containing matrices may provide greater structural support. Excessive reinforcement, however, may restrict cell migration, reduce injectability, or impair matrix remodeling.

### 6.1 Matching degradation kinetics with vascular and bone-formation dynamics

Hydrogel remodeling should match the changing requirements of vascular organization and skeletal tissue formation. Excessively rapid degradation may compromise structural integrity before vascular networks and extracellular matrix become stable; overly slow degradation may restrict endothelial invasion, cell migration, matrix remodeling, and mineral deposition. Cell-mediated MMP proteolysis can generate space for MSC migration, endothelial sprouting, extracellular matrix deposition, and mineral accumulation. Lutolf et al. showed that cell-mediated proteolytic degradation enables 3D cell migration and tissue-like remodeling (Lutolf et al., 2003), but this was not performed in a bone organoid model. For bone organoid systems, a temporally changing degradation profile may be advantageous: early matrix remodeling may support endothelial migration and primitive vascular-network formation, whereas subsequent matrix persistence provides structural support during osteogenic matrix deposition and mineralization. This process is overlapping rather than strictly sequential (Kusumbe et al., 2014; Kanczler and Oreffo, 2008). Photodegradable systems may have limited applicability in large or deeply implanted constructs because of light-penetration constraints (Kloxin et al., 2009). No generally applicable degradation half-life can be recommended; future studies should report degradation kinetics together with residual modulus, mesh size, factor-release profiles, endothelial-network formation, mineral deposition, and organoid viability.

### 6.2 Stress relaxation and cell-state regulation

Stress relaxation, the time-dependent dissipation of stress under constant deformation, can regulate cell spreading, traction-force generation, migration, matrix remodeling, and lineage-associated behavior. Chaudhuri et al. showed that faster stress relaxation promoted MSC spreading, proliferation, osteogenic differentiation, matrix deposition, and bone formation in related models (Chaudhuri et al., 2016), but this was not a bone organoid model. In bone organoid engineering, faster matrix relaxation may facilitate endothelial sprouting, stromal-cell organization, and osteogenic activity when combined with suitable biochemical cues; slower-relaxing matrices may maintain rounded cell morphology and could be useful for chondrogenic or early endochondral stages (Cameron et al., 2011). These effects are context-dependent and cannot be predicted from relaxation time alone. A temporally evolving viscoelastic environment may be useful: a relatively remodelable matrix during early cellular and vascular organization, followed by sufficient matrix stability during osteogenic maturation and mineral deposition. The optimal stress-relaxation time has not been established; future studies should report both initial modulus and full stress-relaxation curve.

### 6.3 Release kinetics and signaling coordination

Release kinetics determine local concentration, exposure duration, and timing of intracellular signaling. Bolus delivery may generate short-lived high-concentration signals; sustained, delayed, or temporally biased release may better coordinate overlapping biological processes. Richardson et al. demonstrated that polymeric systems can generate distinct release profiles for different growth factors (Richardson et al., 2001). Sequential or temporally controlled growth-factor delivery has been associated with improved vascularization and bone regeneration in related models (Kempen et al., 2009; Patel et al., 2008; Richardson et al., 2001; Shi Y. et al., 2025), but these findings are indirect evidence for bone organoid engineering. The optimal dosing window depends on factor identity, dose, binding affinity, hydrogel composition, release mechanism, cell source, and model size. Excessive or premature BMP-2 exposure may increase risk of abnormal mineralization or ectopic bone formation; insufficient angiogenic stimulation may limit nutrient transport and tissue maturation (James et al., 2016). HIF-related signaling can promote angiogenic–osteogenic coupling (Wang et al., 2007; Schipani et al., 2009); BMP-2 is a well-established osteoinductive signal (Urist, 1965; Wozney et al., 1988). Dynamic hydrogels may establish an early angiogenic bias and progressively enhance osteogenic signaling, rather than reproducing a universally fixed two-stage pathway. Actual release profiles are influenced by diffusion, swelling, degradation, factor binding, and cell-mediated remodeling (Huebsch et al., 2014; ter Haar, 2007; Li and Mooney, 2016); future studies should report factor loading, initial burst release, cumulative release, release duration, local concentration, and corresponding vascular and osteogenic outcomes under the same conditions.

### 6.4 Mechanical strength and host-tissue integration

Hydrogel stiffness and mechanical strength influence organoid morphology, matrix deposition, structural maintenance, and integration with host bone. Mechanically insufficient matrices may deform or collapse; excessively stiff or poorly remodelable matrices may restrict cell infiltration, vascular ingrowth, and endogenous matrix deposition. Mechanical mismatch at the graft–host interface may adversely affect load transfer and tissue integration. Studies on 2D substrates demonstrated that matrix elasticity can influence MSC lineage specification; subsequent work showed that matrix mechanics interact with integrin engagement, ligand presentation, and degradability. Related bone-regeneration studies suggest that hydrogel mechanics must be balanced with vascular infiltration and osteogenic differentiation. Larger or load-bearing defects may require reinforcement with calcium phosphate, nanohydroxyapatite, or bioactive glass, but excessive mineral loading can reduce injectability, cell viability, and matrix homogeneity. The appropriate mechanical range for bone organoids cannot currently be defined by a single modulus or strength value; reported properties vary with polymer concentration, crosslinking method, hydration state, testing mode, strain rate, and mineral content. Successful integration depends on the dynamic balance among initial modulus, stress relaxation, degradation, tissue formation, mineral deposition, and interfacial bonding. Future studies should report mechanical properties at multiple time points and relate them to vascularization, mineral density, defect bridging, and host integration.

## 7 Conclusions and perspectives

Dynamic hydrogels provide a promising framework for engineering vascularized bone organoids beyond the limitations of static matrices. The central design principle is not simply to combine vascular and osteogenic components, but to regulate their spatial and temporal interaction. Programmed degradation, tunable viscoelasticity, stimuli responsiveness, and temporally biased biochemical presentation may help reproduce important features of native bone development, in which angiogenic and osteogenic processes overlap but vary in relative contribution over time.

The evidence base remains heterogeneous. Direct evidence from bone organoid models is currently limited; many mechanistic insights derive from MSC cultures, vascularized bone-regeneration constructs, cartilage or endochondral models, wound-healing studies, and general hydrogel systems. Strategies such as degradation-mediated factor release, stress-relaxing networks, piezoelectric composites, and modular microsphere assemblies should be interpreted according to their evidence status rather than as equally validated bone-organoid platforms.

Several challenges remain. First, many current systems use simplified models and limited cell populations. Future studies should incorporate endothelial cells, perivascular cells, osteoprogenitors, immune cells, and, where appropriate, osteoclast-lineage and hematopoietic populations to reproduce the vascular, skeletal, marrow, and remodeling components of the native niche. Cell sourcing will be an important determinant of reproducibility and translational relevance. Induced pluripotent stem cell (iPSC)-derived endothelial and skeletal-lineage cells may facilitate patient-specific modeling and reduce immunogenicity, but they introduce donor-to-donor variability, differences in reprogramming quality, incomplete differentiation, and limited expansion capacity. Primary bone marrow or adipose-derived mesenchymal stromal cells provide greater physiological relevance but are limited by donor age, batch variability, and senescence. Endothelial colony-forming cells or HUVECs remain convenient but may not fully recapitulate bone-specific endothelial phenotypes. Future studies should therefore report cell source, passage number, differentiation efficiency, and key marker profiles, and should validate findings in at least two independent donor lines where possible.

Second, quantitative relationships between hydrogel parameters—initial modulus, stress-relaxation time, degradation half-life, mesh size, ligand density, and factor-release duration—and biological outcomes remain poorly defined. Although representative numerical values are available from selected organoid-like and related hydrogel studies (Table 4), differences in material composition, testing protocols, and biological models currently prevent the definition of universal design windows. Universal numerical thresholds cannot yet be established; standardized reporting (Table 3) and time-resolved measurements are necessary to develop predictive structure–function relationships.

Third, scalability and clinical translation require further attention. Injectable, modular, and biofabrication-compatible systems are promising, but their mechanical integrity, vascular perfusability, degradation behavior, immunological compatibility, and long-term stability in large or load-bearing defects must be rigorously validated. Future studies should distinguish organoid formation from functional vascularization, mineralization, host anastomosis, and mechanical regeneration. Translation of dynamic hydrogel-based bone organoids into clinical grafts faces additional barriers beyond biological performance. Sterilization methods (e.g., gamma irradiation, ethylene oxide) may alter hydrogel mechanics and growth factor bioactivity; endotoxin levels must be controlled for all components, particularly those derived from natural polymers such as gelatin, alginate, and silk fibroin. Batch-to-batch variability in polymer molecular weight, degree of substitution, and crosslinking efficiency affects reproducibility. Recombinant growth factors such as BMP-2 and VEGF are expensive and may require supraphysiological doses in clinical settings, raising safety and cost concerns. Cryopreservation, shipping, and shelf-life stability of cell-laden constructs remain unresolved. Finally, a cell-laden organoid combined with a synthetic or semi-synthetic hydrogel is likely to be regulated as a combination product, requiring demonstration of safety, potency, and consistency. These practical constraints should be considered in parallel with biological design.

Looking forward, convergence of dynamic hydrogels with advanced biofabrication, organoid biology, and computational approaches may accelerate development of more adaptive bone organoid systems. A future workflow could integrate artificial intelligence (AI)-guided material design, real-time imaging, adaptive organoid culture, and patient-specific modeling. Machine-learning approaches may help identify relationships among polymer chemistry, degradation kinetics, mechanical behavior, cell composition, and organoid outcomes, but such applications remain exploratory and depend on standardized datasets, interpretable models, robust sensing methods, and validation across independent platforms. AI should be regarded as a potential design and optimization tool rather than an established method for autonomous control of angiogenic–osteogenic coupling. Integrating live-cell imaging with computational analysis may enable feedback-based adjustment of culture conditions, but closed-loop systems remain at an early conceptual stage.

Although clinical translation remains challenging, the principles discussed provide a framework for developing bone organoid systems that are not only structurally biomimetic but also functionally regenerative. Further progress requires direct validation in multicellular bone organoid models, standardized quantitative characterization, reproducible vascularization, and rigorous assessment of host integration. With continued interdisciplinary effort, dynamic hydrogel platforms may eventually contribute to vascularized bone repair and personalized skeletal medicine, although their clinical efficacy remains to be demonstrated.
